# Food Security in Brazil: Evaluation of the Effectiveness of Community Restaurants in the North and Northeast Regions

**DOI:** 10.3390/ijerph22020315

**Published:** 2025-02-19

**Authors:** Mateus Santana Sousa, Rita de Cássia Akutsu, Calliandra Maria de Souza Silva, Camila Silveira Silva Teixeira, Izabel Cristina Rodrigues da Silva

**Affiliations:** 1 Graduate Program in Food, Nutrition and Health, Nutrition School, Federal University of Bahia, Salvador 40110-909, Brazil; mattheus_sousa@outlook.com (M.S.S.); rita.akutsu@gmail.com (R.d.C.A.); 2 Department of Nutrition, Faculty of Health Sciences, Campus Universitario Darcy Ribeiro, University of Brasília, Brasilia 70910-900, Brazil; 3 Graduate Program in Health Sciences and Technologies, Faculty of Ceilândia, University of Brasília, Federal District, Brasília 72220-900, Brazil; cdssilva@gmail.com; 4 Medical Science Center, Federal University of Pernambuco, Recife 50670-420, Brazil; camila.steixeira@ufpe.br

**Keywords:** evaluation, effectiveness, Community Restaurants, food and nutritional security

## Abstract

Community Restaurants (CRs), part of the Brazilian government’s Popular Restaurant Program (PRP), provide free and/or affordable nutritionally balanced meals as an essential strategy to meet the most vulnerable population’s basic needs and rights. This cross-sectional study evaluated the effectiveness of these CRs’ proposed objectives in cities with ≥100,000 inhabitants in Brazil’s northern and northeastern regions—Brazil’s most vulnerable and historically unequal regions. Effectiveness was assessed using a CR evaluation matrix-based indicator system to classify CRs as “not very effective”, “effective”, or “very effective”. Among the 94 CRs assessed (north: n = 23, 24.5%; northeast: n = 71, 75.5%), most were classified as “effective” or “very effective”, except for the northern states of Amapá and Tocantins, whose only CR was rated as “not very effective” and the northeastern state of Sergipe, which had a PR considered “not very effective”. State-operated CRs predominated (north: 82.6%; northeast: 76.1%) and primarily operated Monday to Friday (north: 87.0%; northeast: 59.2%), with 35.2% of the northeast CRs operating daily, serving lunch as the main meal (100%). Average daily meal counts were 486.5 (north) and 926.9 (northeast), and the average meal offering time was from 10:55 am to 1:21 pm in the north and from 10:35 am to 2:00 pm in the northeast region, with costs averaging USD 0.27 and USD 0.20, respectively. All CRs employed a nutritionist as a technical manager responsible for menu planning and demonstrated compliance with essential infrastructure criteria, including regular waste collection, water supply, and proximity to public transport. Most were in areas with sanitary sewage coverage (north: 91.3%; northeast: 98.6%) and had monitoring mechanisms (91.3% north; 94.4% northeast) and prioritization systems for vulnerable populations (north: 73.9%; northeast: 80.0%). These findings indicate that CRs in these regions effectively strive to address food security goals, demonstrating tangible outcomes that benefit society.

## 1. Introduction

The evaluation of public policies represents an essential part of evidence-based decision-making and is considered a recommended practice for public administration. Such an evaluation must be carried out as a systematic, permanent, integrated, and institutionalized process, with the basic premise of identifying ways to improve public action processes, results, and management [1,2,3,4,5,6,7]. One form of assessment can be based on the effectiveness criterion, which refers to the results achieved by the policies, programs, and projects established and implemented by the public administration and their ability to effectively achieve the proposed objectives for the benefit of society [4,5,6,7,8]. This principle was adopted by the Brazilian government, based on the Federal Constitution of 1988 [9], and is in line with the management administration model, which stresses results-oriented control, which implements modern management tools, over bureaucratic processes [10].

Evaluation must be carried out on an ongoing basis and integrated into the public policy cycle, which involves stages of planning, execution, and budgetary/financial control. The effective use of the evaluation results enables the adoption of measures capable of guiding public administrators, with the primary objective of improving public policies’ implementation [2,5,6,7].

Public food policy evaluations stand out for their concern regarding ensuring continuous food access and reducing food and nutritional insecurity levels in society. Adequate nutrition is a basic requirement for the promotion and protection of health, being recognized as a determining and conditioning factor in the health situations of individuals and communities [11,12,13,14], and is one of the constitutional guarantees included among the social rights of the Brazilian Federal Constitution [9].

The Popular Restaurant Program (PRP) is part of one of the Brazilian government’s social protection policies, which aims to offer nutritionally balanced meals either free of charge, at affordable prices, or both, especially for populations experiencing food insecurity, social vulnerability, or both. In addition to guaranteeing food and nutritional security (FNS), these Community Restaurants (CRs; “restaurantes populares” in Brazilian Portuguese) are part of a public policy aimed at promoting healthy eating habits and valuing regional culture/food [15,16,17,18,19,20,21]. The CRs offer between 500 and more than 2000 meals daily, at lunchtime, every week (Monday to Friday) [22,23,24] and are distributed across the five Brazilian regions linked to the National Food Assistance Program. Due to their different geographic regions, each state has its own gastronomic identity, full of unique flavors and stories. Hence, the meals served in each CR unit incorporate the local food culture, presenting regional preparations on the menu served to its users.

As stated, CRs are an essential strategy to meet the basic needs and rights of the population, especially in the north and northeast, Brazil’s most vulnerable and historically unequal regions. These regions had the lowest average monthly household income per capita in 2023 (USD 224.83 and 197.89, respectively), compared to the national average (USD 319.12) and other Brazilian regions (southeast: USD 386.29; central-west: USD 380.25; and south: USD 374.20) [25]. They also showed lower access to food and the country’s highest rates for food and nutritional insecurity (39.7% and 38.8%, respectively) [26].

Although CRs are fundamental to guaranteeing FNS in all its dimensions and the broader reach of public policies, evaluations of these facilities are still incipient. Most of the time, they are only inspected by regulatory bodies, such as the Ministry of Transparency, the Comptroller General of Brazil, the Federal Court of Auditors, and the State and Municipal Court of Auditors, which monitor and control the funds allocated, without evaluating whether these funds are being directed to comply with the program’s norms/objectives or the impact of its actions. Therefore, given the significance of these facilities, especially in Brazil’s northern and northeast regions, this study aimed to determine the effectiveness of the proposed objectives for Community Restaurants (CRs) in cities with a population of ≥100,000 in Brazil’s northern and northeastern regions.

## 2. Materials and Methods

### 2.1. Study Design and Population

This cross-sectional study used Community Restaurant (CR) data from Brazil’s Popular Restaurant Program (PRP). For this study, all CRs in cities with an estimated population of ≥100,000 inhabitants in states from Brazil’s northern (Acre, Amapá, Amazonas, Pará, Rondônia, Roraima, and Tocantins) and northeastern (Alagoas, Bahia, Ceará, Maranhão, Paraíba, Pernambuco, Piauí, Rio Grande do Norte, and Sergipe) regions were considered eligible. Therefore, this study included data from 90 cities based on the latest 2022 population census by the Brazilian Institute of Geography and Statistics (IBGE) [27].

### 2.2. Data Source and Collection

**Figure 1:**
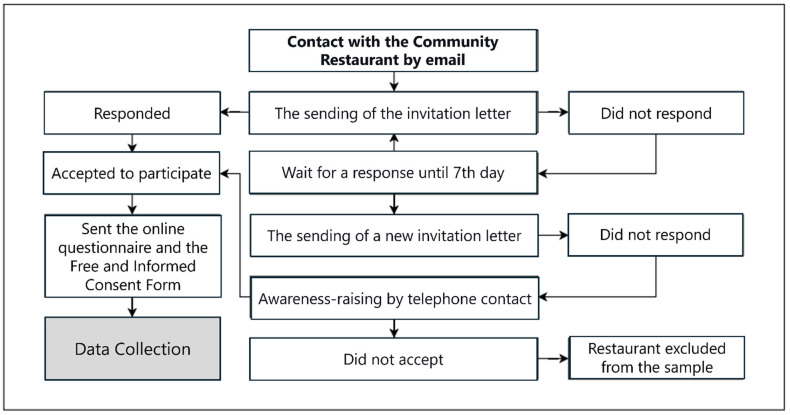
Description of the data collection process, Brazil, 2024.

Initially, city halls and/or state governments were contacted to inform them of the survey and verify the CR units’ existence in their localities. Telephone and email contacts were screened, with the aim of future contact with the management of the respective municipality’s development and social assistance departments. Through this preliminary survey, 106 municipal and state-operated CR units—28 units (26.4%) from the north and 78 units (73.6%) from the northeast regions—were considered eligible from cities with ≥100,000 inhabitants, according to the implementation and location criteria of the Brazilian Federal Government’s Manual for Implementing Popular Restaurants [23], which guides the implementation of CR units in the national territory. In order to collect data, a letter inviting CR managers to participate in the survey was sent, and those who agreed to participate in the study were asked for the technical managers’ telephone numbers and email addresses so that the online questionnaire could be sent (Figure 1).

Data were collected between April and June 2024 by adapting the evaluation and judgment matrix to an online questionnaire that the technical managers or PR managers self-completed using the Google Forms^®^ tool. A Free and Informed Consent Form (FICF) was also priorly applied to all who agreed to participate in the survey.

### 2.3. Instruments and Classification

To evaluate the effectiveness of CRs, we used the indicators from the validated Community Restaurants evaluation matrix developed by Oliveira et al. [28,29,30]. The matrix comprises 2 dimensions, 6 sub-dimensions, 24 indicators, and 29 measures. The methodology proposes direct observation and monitoring of events to understand the behavior of the object of study. This flexible technique allows for the comparison of theoretical information with reality [29]. This classification method is standard in the health evaluation field [31,32].

All indicators in the CRs evaluation matrix have the same degree of importance, with each being able to be classified as “good”, “regular”, or “bad”. For facilitating the value judgment, a score of 10 was attributed to the indicators classified as “good”, 5 points to those classified as “regular”, and 0 points to those classified as “poor” (Table 1).

The CR assessment was classified using the average of the indicators. When the indicator (see Data Analysis section for the considered indicator) has only two evaluation measures, a value judgment of “good” was assigned when the score was ≥5 and “bad” when <5. For the remaining indicators, sub-dimensions, dimensions, and the final CR assessment, the value judgment was classified as “good” when it was >7.0, “regular” when it fell between ≤7 and ≥5.0, and “bad” when it was <5.0.

### 2.4. Data Analysis

We used the indicator system method developed by Januzzi and Patarra (2006) [33] to assess the effectiveness of the CR, i.e., its ability to achieve objectives and intended results. The indicators considered were availability, human resources, financial resources, meal price, coordination with other programs and FNS, waste management, quality evaluation and monitoring, location, physical structure, maintenance, users, prioritization of specific populations, menu planning, food safety, regional foods and recipes, preference for regional ingredients, number of meals served, user satisfaction, education in food and nutrition and FNS, promotion of other social assistance initiatives, the local food and nutrition security index, socialization activities, intersectoral actions, and visibility of the restaurants. The restaurants were classified, according to the criteria proposed by Hartz and Silva (2005) [6], as “not very effective” when they presented an evaluation percentage lower than 50%, “effective” between 50 and 70%, and “very effective” if over 70% (Table 2).

Differences between proportions were tested using Pearson’s chi-square or Fisher’s exact test (for categories with a number of observations < 5). The effectiveness of the CRs was estimated using the prevalence ratio (PRr). After data analysis, outliers were excluded so as not to compromise the sample. Descriptive analysis was performed to characterize the sample, and *p* < 0.05 was considered statistically significant. Prevalence ratios, 95% confidence intervals (95% CI), mean/standard deviation, and the median/interquartile range were calculated for continuous variables. A comparative analysis was carried out between the results obtained in the two geographical regions, between the states of the regions, and between the municipalities. Data were analyzed using the Statistical Package for Social Science for Windows (SPSS 23.0, United States of America).

### 2.5. Ethical Aspects

This study was approved under opinion No. 5.613.160 by the Ethics Committee of the School of Nutrition of the Federal University of Bahia under Resolution 466/2012 of the National Ethics and Research Commission [34]. No personally identifiable information was included in the dataset used for analysis.

## 3. Results

**Figure 2:**
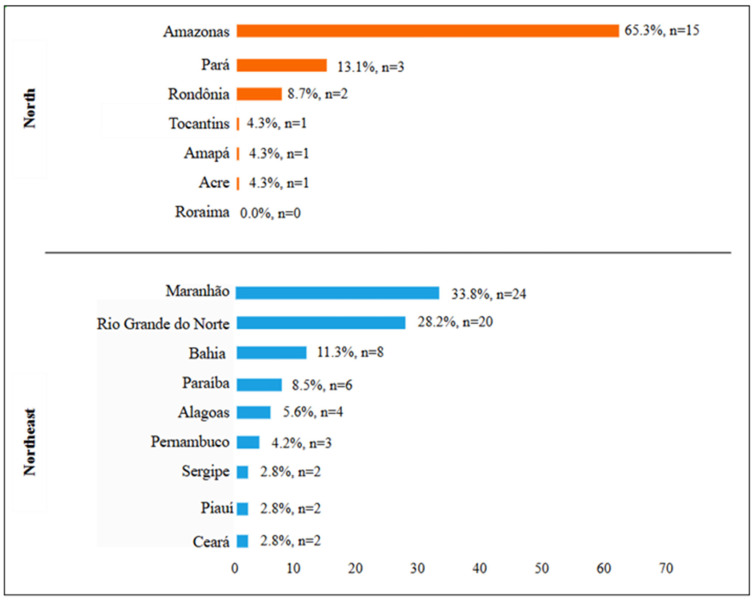
Distribution and percentage of Community Restaurants according to the states of the north and northeast regions of Brazil, 2024.

Of the total 106 municipal and state-operated Community Restaurant (CR) unit sample invited, we evaluated 94 CRs located in cities with a population of ≥100,000 inhabitants in the north (n = 23; 24.5%) and northeast (n = 71; 75.5%) regions that agreed to the survey. In the north, most CRs were concentrated in the state of Amazonas (65.3%; n = 15) and in the northeast in the states of Maranhão (33.8%; n = 24) and Rio Grande do Norte (28.2%; n = 20) (Figure 2).

In the north and northeast, respectively, most CRs are administered by the state (82.6%; 76.1%) and operate from Monday to Friday (87.0%; 59.2%), with 35.2% of CRs in the northeast operating daily (Table 3). They serve an average of 486.5 meals (±290.3; median = 900; Q1 = 340-Q3 = 500) in the north and 926.9 (±446.0; median = 900; Q1 = 560-Q3 = 1100) in the northeast. Lunch is the meal primarily served in 100.0% of CRs in these regions, and the average time for offering this meal is from 10:55 am to 1:21 pm in the north and from 10:35 am to 2:00 pm in the northeast. The average cost of lunch is USD 0.27 (±0.16) in the north and USD 0.20 (±0.11) in the northeast (Table 3).

All CRs (100.0%) in the north and northeast have a nutritionist in charge of menu planning, with the nutritionist also being the professional responsible for this planning (Table 1). Likewise, all of them are in areas with regular garbage collection, water supply, and proximity to public transport (Table 3). Most of the restaurants in these regions are in areas covered by sanitary sewage (91.3% north; 98.6% northeast), have evaluation/monitoring mechanisms (91.3% north; 94.4% northeast), and differentiated/prioritized service for people in situations of social vulnerability and food and nutritional insecurity (73.9% north; 80.0% northeast) (Table 3).

The number of outliers in the sample was small. Nevertheless, it was crucial to remove them as atypical values could artificially alter their prevalence by overestimating or underestimating them.

The effectiveness assessment reveals that most CRs in the north were considered “effective” or “very effective”, except for the states of Amapá and Tocantins, whose only CR was rated as “not very effective’”. In the northeast, most states also had their CRs classified as “effective’” or ”very effective”, and only Sergipe had a PR considered “not very effective” (Table 4).

## 4. Discussion

Most of the Community Restaurants (CRs) evaluated in the north and northeast were considered “effective” or “very effective”, with the only differing restaurants being in the northern states of Amapá and Tocantins, which had their only CR evaluated as “not very effective”, and in the northeastern state of Sergipe, where one CR was considered “not very effective”. These data indicate that Brazil’s Popular Restaurant Program (PRP) in these regions has strived to meet its targets and present society with tangible results that benefit the population. This endeavor is essential to ensuring that public resources are handled responsibly and that government action actually meets society’s needs [4,7,35,36,37]. Implementing corrections to the planning and service provided in the CR units that demonstrated suboptimal effectiveness in achieving the proposed objectives is also crucial.

Effectiveness in public management is essential, as it seeks an effective administration capable of offering quality services to society [4,7]. In the current context, especially in Brazil, where public policies receive large amounts of funding, society has increasingly higher expectations regarding public services, and government institutions must be able to meet these demands effectively [38,39]. Thus, when people see that the programs arising from public policies work effectively and improve society, they tend to support and participate in democratic processes [36,37].

The main objective of a CR’s existence is access to food. According to data made available by the Food and Agriculture Organization of the United Nations (FAO), around 12% (928 million) of the world’s population is in a situation of severe food insecurity, i.e., living with hunger and consequently without regular and permanent access to food [40]. In Brazil, the data indicate that more than 70 million Brazilians are in moderate or severe food insecurity, and 10 million are malnourished [41]. All these lines of evidence demonstrate a need for social policies to serve the population.

Access to food is a fundamental human right internationally recognized by the Universal Declaration of Human Rights [42] and the Federal Constitution of Brazil [9]. Its premise is to ensure that all people have access to food of suitable quality, in sufficient quantity, on a permanent basis, without compromising access to other essential needs. This right is directly related to food and nutritional security (FNS), which is the duty of the state and the responsibility of civil society as a whole to promote [9,42,43]. Accordingly, it is up to the state to develop an FNS policy by establishing permanent food policies and programs, considering national sovereignty and the Federal Constitution, and structuring it through health promotion principles and strategies [43,44,45].

In Brazil, public policies, such as the Food Acquisition Program (PAA) [46], the Popular Restaurants Program (PRP) [23], and the National School Feeding Program (PNAE) [47], are examples of initiatives aimed at guaranteeing this right, promoting access to healthy food, and encouraging family farming. As previously mentioned, a significant portion of the Brazilian population lives without minimum access to food, as defined by the PRP [48]. Thus, the effective presence of CRs in vulnerable regions, such as the north and northeast of the country, contributes to mitigating food insecurity by promptly meeting this immediate and urgent need. However, this support does not change the beneficiaries’ vulnerability conditions.

Among the data analyzed, it was possible to see that three states, one in the north (Amazonas) and two in the northeast (Maranhão and Rio Grande do Norte), had the highest concentrations of CRs in the sample. Currently, the state of Amazonas has 44 CR units distributed in 18 municipalities [49], the state of Maranhão has 175 CR units in 158 municipalities [50], and the state of Rio Grande do Norte has 56 CR units in 34 municipalities [51]. These states stand out for having created, in the last 10 years, a policy of expansion, decentralization, and internalization of anti-hunger programs, strengthening public policy actions in favor of food and nutritional security [52,53]. The state of Maranhão has set itself the goal of having all 217 municipalities equipped with a CR unit by the end of 2026, making it the most extensive food security program in Latin America. The program is a reference in the fight against hunger, serving as an implementation model for other states in Brazil [50].

Maranhão currently has the largest number of CRs in Brazil, surpassing the state of São Paulo, which 23 years ago had the largest and most traditional chain of CRs in the country, with 120 units installed, 75 of which were fixed and 45 were mobile [54,55,56]. Notably, the state of Maranhão has the lowest per capita income in the country (USD 163.18) and one of the highest percentages of food and nutritional insecurity in households (63.3%) [26]. As such, the state administration seeks, through expanding CRs, to reduce food and nutritional insecurity levels and promote qualifications and training.

This structuring of the Community Restaurant Program in the state of Maranhão, as a state government policy, may perhaps explain the disparity in the effectiveness of the CRs between states, such as the states of Amapá and Tocantins, which only have CR units in their capitals. Successful experiences like this could be used by other federal entities to achieve the best effectiveness rates, which, in an ideal scenario, may include integrating these initiatives with partnerships with other sectors, such as public health services and social assistance, and other public food policies, such as the purchase of food from family farming, the promotion of food and nutritional education, income transfer programs, and access to jobs aimed at this low-income population [28,57].

The Manual for Implementing Community Restaurants [23] also recommends that the local town halls carry out CR management in partnership with various municipal departments. In a study carried out in three states in the southern region of the country (Paraná, Santana Catarina, and Rio Grande do Sul), of the 30 participating CRs, 57% had municipal management [30], which differs from our study in which state governments mostly carry out the management of these facilities. This change in profile may be due to the fact that state governments have a greater capacity to create and maintain public policies and programs in northern and northeastern states. This greater capacity includes greater financial power, technical skills, and administrative structure compared to local city governments.

Both northern and northeastern regions’ CRs operated from Monday to Friday, with lunch being the priority meal and an average service time of three hours for the public. These results are in line with the guidelines for implementing CRs, which express that they must operate at least five days a week during lunchtime [23], and are corroborated by the evidence of Sousa et al. (2021) [20] and the Brazil Public Opinion Survey (2005) [58]. Unlike the recommended, which establishes an estimated minimum production of one thousand meals per day, the CRs evaluated were below the average (north: 486.5; northeast: 926.9), which suggests a change in the supply profile, considering that a portion of the CRs created in recent years has an average daily supply of 500 meals.

Even though the evaluation occurred in cities with a population of ≥100,000, the decentralization of CR units to neighborhoods far from the city center may partly explain the observed service profile. These new location configurations allow neighborhoods with large populations to receive CR units in addition to urban centers. Furthermore, all the CRs evaluated reported being close to places with public transportation access, as the PRP recommended [23].

Hence, the CRs evaluated show the potential to fulfill their objective of ensuring adequate food for users, especially for those for whom the food served may be the only alternative to having at least one full meal per day [59,60]. Despite these findings and the fact that some CRs operate daily, we emphasize the need for all CRs to serve the public daily, including weekends and public holidays, and offer complimentary breakfast and dinner meals.

Regarding the amount charged for the meal served at lunchtime, the regions evaluated presented the lowest average amounts charged, with the northern region being USD 0.27 and the northeastern being USD 0.20 compared to the national average of USD 0.34 and other Brazilian regions (central-west: USD 0.35; southeast: USD 0.40; and south: USD 0.39) [61]. One of the main objectives of CRs is to offer free or affordable meals without the amount paid compromising the income of their users [18,21,62]. Studies carried out with CR users in Brazil [16,58,63,64,65,66] revealed that they consider the amount paid for meals to be affordable. According to these studies, approximately 98% of users believe that the price charged is affordable, and of these, 84% consider that the value of meals represents a low or very low cost in their personal budgets [16,58,63,64,65,66].

Other significant findings comprised the inclusion of a nutritionist as the technical manager and menu planner, with the nutritionist being the professional responsible for this planning. According to the Federal Council of Nutritionists (CFNs), the nutritionist should be the professional responsible for planning and supervising the preparation of menus, ensuring that meals are nutritionally balanced and meet the specific needs of consumers [67]. The presence of this professional in the Food and Nutrition Units (FNUs) is fundamental, as it is of utmost importance to guarantee the quality and safety of the food offered. However, the literature provides evidence that this requirement is not always fulfilled. Júnior et al. (2020) [68] found that only 55% of FNUs in private companies, hotels, and hospitals had a technical manager, becoming a preponderant factor in outbreaks of food-related diseases. Furthermore, to ensure nutritionally balanced food [24,69], the CFN’s Resolution No. 380 recommends that companies providing collective food, self-managed food services, commercial restaurants, and similar establishments must have a nutritionist responsible for technical matters, as well as the diversity and complexity of the menus offered [70].

All CRs had regular garbage collection and water supply, and most of these restaurants are in regions covered by sanitary sewage systems (91.3% north; 98.6% northeast). These services prevent the proliferation of pests and food contamination, as accumulated waste can attract insects and rodents, which are disease vectors and can compromise food quality [71,72]. These infrastructure services in FNUs are essential to ensure food safety, protect public health, preserve the environment, and maintain compliance with health standards.

Finally, the evaluated CRs reported having evaluation/monitoring mechanisms (91.3% north; 94.4% northeast) and differentiated/prioritized service for those experiencing social vulnerability and food and nutritional insecurity (73.9% north; 80.0% northeast). The central evaluations were user satisfaction surveys regarding the menu offered, a user profile characterization, and their food and nutritional insecurity level. The CR management, local and state councils, and university centers all undertake these evaluations. Even if deemed one-time events, these assessments can be used to guide and improve service quality.

Characterizing the potential public, often comprised of people who travel and/or work in the CR region, is critical to creating a socioeconomic user profile [23,24]. This information can serve as a basis for creating strategies to prioritize people experiencing social vulnerability and food and nutritional insecurity. International programs similar to Brazilian CRs have also created strategies to prioritize this target audience, such as reserving a percentage of access to the CRs, free meals, and differentiated opening hours [73,74,75,76,77,78,79,80,81,82]. These strategies aim to ensure that the target audience can access the CRs and, consequently, their meals.

This study aimed to contribute to the PRP and its managers, adding scientific, academic, and social knowledge and cooperation between researchers from different centers studying food and nutritional security. Nevertheless, the study has limitations due to its cross-sectional design; for example, the data were obtained at a single time point. Furthermore, the online data collection may have prevented the researchers from seeing the research object. Using the CR evaluation matrix may have also limited the inclusion of critical food insecurity indicators and the option of integrating other indicators and measures in the evaluation model for a more complete and specific measurement of CR effectiveness. As a result, new studies are essential to promote future improvements in evaluation methods. Another limitation is the impossibility of comparing and discussing the results obtained with solutions applied in other countries since none of them has a program with objectives and rules similar to those of the Brazilian PRP.

## 5. Conclusions

This study evaluated Community Restaurants’ effectiveness in achieving their proposed objectives in Brazil’s northern and northeastern regions. The data indicate that most of the restaurants in the regions evaluated were classified as “effective” or “very effective” in meeting their goals, enabling them to present tangible results to society that benefit the population. Presenting the effectiveness of these facilities is fundamental to ensuring that public policies and programs are not just good intentions but concrete actions whose fundamental premise is to promote social well-being and sustainable development.

Among the relevant findings that emerged from this study, those related to the low price of the meal offered, proximity to public transport, presence of a nutritionist as a technical manager, menu planning, availability of essential services (regular garbage collection and availability of sewage system and running water), presence of evaluation/monitoring mechanisms, and the prioritization of vulnerable populations and those in situations of food and nutritional insecurity stand out.

These results indicate that Community Restaurants have sought to promote access to food effectively through their actions. Nonetheless, we emphasize the importance of offering this food daily and, if possible, in at least two meals per day. The evidence suggests that these facilities collaborate positively in terms of access to reduce food and nutritional insecurity among the Brazilian population. This fact has significant implications for the area of food and nutritional security. New studies that focus on evaluating Community Restaurants while incorporating longitudinal or mixed methods are needed to understand these facilities better and address the limitations identified in our research. For instance, our cross-sectional design did not allow for causality or longitudinal trend assessments, and self-reported data from questionnaires could introduce reporting bias as the researchers did not directly access the research object. These findings advance public food and nutrition policy discussions and contribute to the literature and public managers’ management.

## Figures and Tables

**Table 1:** The Community Restaurants evaluation matrix indicator scores, Brazil, 2020 [28,29,30].

Classification	Score
Good	10 points
Regular	5 points
Bad	0 points

**Table 2:** Criteria for evaluating effectiveness in health programs and systems [6].

Percentage	Classification	Effectiveness
<50%	Low	Not very effective
50 to 70%	Adequate	Effective
>70%	High	Very effective

**Table 3:** Profile of Community Restaurants in Brazil’s north and northeast regions, 2024.

Variables	North (n = 23)			Northeast (n = 71)		
	n	%	95% CI	n	%	95% CI
**Type of management**						
State	19	82.6	60.3–93.7	54	76.1	64.6–84.7
Municipal	4	17.4	6.3–39.7	17	23.9	15.3–35.4
**Days of operation**						
Daily	2	8.7	2.0–30.6	25	35.2	24.9–47.1
Monday to Friday	20	87.0	64.9–96.0	42	59.2	47.2–70.1
Weekdays and Public Holidays	1	4.3	0.5–27.5	4	5.6	2.1–14.3
**Number of meals served**						
Mean/Standard Deviation	486.5	-	290.3	926.9	-	446.0
Median/Interquartile Range	400	-	340–500	900	-	560–1100
**Meals served (isolated)**						
Breakfast	1	4.4	0.5–27.5	17	23.9	15.3–35.4
Lunch	23	100.0	-	71	100.0	-
Dinner	-	-	-	38	53.5	41.7–64.9
**Meals served (combined)**						
Breakfast and Lunch	1	4.4	0.5–27.5	4	5.6	2.1–14.3
Lunch and Dinner	-	-	-	22	31.0	21.2–42.8
Breakfast, Lunch, and Dinner	-	-	-	13	18.3	10.8–29.2
**Opening hours (lunch)**						
* Start time*						
Mean/Standard Deviation (hh:mm)	10:55	-	00:20	10:35	-	00:14
Median/Interquartile Range (hh:mm)	11:00	-	11:00–11:00	10:30	-	10:30–11:00
* End time*						
Average/Standard Deviation	13:21	-	00:39	13:54	-	00:20
Median/Interquartile Range	13:00	-	13:00–14:00	14:00	-	14:00–14:00
**Value of meal (lunch)**						
Average/Standard Deviation (USD)	0.27	-	0.16	0.20	-	0.11
Median/Interquartile Range	1	-	1–2	1	-	1–1
Free	0	0.0	-	4	5.6	2.1–14.3
Between USD 0.17 and 0.34	19	82.6	60.3–93.7	63	88.7	78.8–94.3
Greater than USD 0.34	4	17.4	6.3–39.7	4	5.6	2.1–14.3
**Presence of a technical manager**						
Yes	23	100.0	-	71	100.0	-
No	-	-	-	-	-	-
**Menu planning**						
Yes	23	100.0	-	71	100.0	-
No	-	-	-	-	-	-
**Who plans the menus**						
Nutritionist	23	100.0	-	71	100.0	-
Other	-	-	-	-	-	-
**Regular garbage collection**						
Yes	23	100.0	-	71	100.0	-
No	-	-	-	-	-	-
**Sanitary sewage**						
Yes	21	91.3	69.4–98.0	70	98.6	90.4–99.8
No	2	8.7	2.0–30.6	1	1.4	0.2–9.6
**Pipe water**						
Yes	23	100.0	-	71	100.0	-
No	-	-	-	-	-	-
**Proximity to public transportation**						
Yes	23	100.0	-	71	100.0	-
No	-	-	-	-	-	-
**Assessment/monitoring**						
Yes	21	91.3	69.4–98.0	67	94.4	85.7–97.9
No	2	8.7	2.0–30.6	4	5.6	2.1–14.3
**Prioritization of specific populations**						
Yes	17	73.9	51.4–88.4	56	80.0	67.8–87.9
No	6	26.1	11.6–48.6	14	20.0	12.1–31.2
Unknown	-	-		1	-	

**Table 4:** Classification of Community Restaurants in Brazil’s north and northeast regions in regard to their effectiveness, 2024.

Region/State	N	Classification		
		Not Very Effective	Effective	Very Effective
**North**				
Acre	1	-	-	1 (100.0%)
Amapá	1	1 (100.0%)	-	-
Amazonas	15	-	15 (100.0%)	-
Pará	3	-	2 (66.7%)	1 (33.3%)
Rondônia	2	-	1 (50.0%)	1 (50.0%)
Tocantins	1	1 (100.0%)	-	-
**Northeast**				
Alagoas	4	-	4 (100.0%)	-
Bahia	8	-	8 (100.0%)	-
Ceará	2	-	1 (50.0%)	1 (50.0%)
Maranhão	24	-	21 (87.5%)	3 (12.5%)
Paraíba	6	-	6 (100.0%)	-
Pernambuco	3	-	2 (66.7%)	1 (33.3%)
Piauí	2	-	2 (100.0%)	-
Rio Grande do Norte	20	-	20 (100.0%)	-
Sergipe	2	1 (50.0%)	1 (50.0%)	-

## Data Availability

The original contributions presented in this study are included in the article. Further inquiries can be directed to the corresponding author.
